# Identification of CD34+/PGDFRα+ Valve Interstitial Cells (VICs) in Human Aortic Valves: Association of Their Abundance, Morphology and Spatial Organization with Early Calcific Remodeling

**DOI:** 10.3390/ijms21176330

**Published:** 2020-08-31

**Authors:** Grzegorz J. Lis, Andrzej Dubrowski, Maciej Lis, Bernard Solewski, Karolina Witkowska, Veronika Aleksandrovych, Ewa Jasek-Gajda, Mateusz K. Hołda, Krzysztof Gil, Jan A. Litwin

**Affiliations:** 1 Department of Histology, Jagiellonian University Medical College, 31-034 Kraków, Poland; ewa.jasek@uj.edu.pl (E.J.-G.); j.a.litwin@uj.edu.pl (J.A.L.); 2 Department of Anatomy, Jagiellonian University Medical College, 31-034 Kraków, Poland; andrzej.dubrowski@uj.edu.pl (A.D.); mkh@onet.eu (M.K.H.); 3 Faculty of Medicine, Jagiellonian University Medical College, 31-008 Kraków, Poland; maciej97.lis@gmail.com (M.L.); bsolewski@gmail.com (B.S.); karolinawitkowska96@gmail.com (K.W.); 4 HEART—Heart Embryology and Anatomy Research Team, Jagiellonian University Medical College, 31-034 Kraków, Poland; 5 Department of Pathophysiology, Jagiellonian University Medical College, 31-121 Kraków, Poland; v.aleksandrovych@uj.edu.pl (V.A.); krzysztof.m.gil@uj.edu.pl (K.G.)

**Keywords:** valve interstitial cells, calcification, CD34, PDGFRα, calcific aortic valve disease

## Abstract

Aortic valve interstitial cells (VICs) constitute a heterogeneous population involved in the maintenance of unique valvular architecture, ensuring proper hemodynamic function but also engaged in valve degeneration. Recently, cells similar to telocytes/interstitial Cajal-like cells described in various organs were found in heart valves. The aim of this study was to examine the density, distribution, and spatial organization of a VIC subset co-expressing CD34 and PDGFRα in normal aortic valves and to investigate if these cells are associated with the occurrence of early signs of valve calcific remodeling. We examined 28 human aortic valves obtained upon autopsy. General valve morphology and the early signs of degeneration were assessed histochemically. The studied VICs were identified by immunofluorescence (CD34, PDGFRα, vimentin), and their number in standardized parts and layers of the valves was evaluated. In order to show the complex three-dimensional structure of CD34+/PDGFRα+ VICs, whole-mount specimens were imaged by confocal microscopy, and subsequently rendered using the Imaris (Bitplane AG, Zürich, Switzerland) software. CD34+/PDGFRα+ VICs were found in all examined valves, showing significant differences in the number, distribution within valve tissue, spatial organization, and morphology (spherical/oval without projections; numerous short projections; long, branching, occasionally moniliform projections). Such a complex morphology was associated with the younger age of the subjects, and these VICs were more frequent in the spongiosa layer of the valve. Both the number and percentage of CD34+/PDGFRα+ VICs were inversely correlated with the age of the subjects. Valves with histochemical signs of early calcification contained a lower number of CD34+/PDGFRα+ cells. They were less numerous in proximal parts of the cusps, i.e., areas prone to calcification. The results suggest that normal aortic valves contain a subpopulation of CD34+/PDGFRα+ VICs, which might be involved in the maintenance of local microenvironment resisting to pathologic remodeling. Their reduced number in older age could limit the self-regenerative properties of the valve stroma.

## 1. Introduction

Aortic valve lesion, known as calcific aortic valve disease (CAVD) is the most widespread pathology of the heart valves. It is associated with intravalvular inflammation, calcium and lipid deposition, and overproduction of the aberrant extracellular matrix, resulting in valve sclerosis and consequently, stenosis [1,2]. Discrete calcification occurs very early in this pathology, leading with time to irreversible, substantial calcific degeneration, and sometimes ossification [1,3,4,5]. Theories on mechanisms involved in CAVD development have been evolving through the years, leading to the currently accepted view, that at the tissue level, this pathology is a process actively regulated by various resident and migratory cells (e.g., interstitial or endothelial cells versus inflammatory cells) [1,5,6]. Since the mechanism of this process remains unclear, there is still no effective treatment other than surgical replacement of the defective valve.

Aortic valve (AV) ensures unidirectional blood flow preventing retrograde flow from the aorta into the left ventricle. Anatomically, the aortic valve (AV) is composed of three cusps (leaflets): left coronary cusp (LCC), right coronary cusp (RCC), and non-coronary cusp (NCC), attached to the fibrous ring, forming as a whole, a crown-like structure [7]. Each cusp is covered by endothelial cells and composed of three connective tissue layers: (i) highly collagenous fibrosa, facing aorta lumen, (ii) glycosaminoglycans/proteoglycans-rich spongiosa lying at the middle level, and (iii) elastin-rich ventricularis, facing the ventricular cavity. Under normal conditions, cusps are thin, pliable, avascular, autonomically innervated structures coated by valve endothelial cells (VECs), and populated mainly by valve interstitial cells (VICs) [8,9]. VICs are located in each layer of the aortic valve [10] and synthesize extracellular matrix components, including collagen, elastin, and glycosaminoglycans, hence they functionally resemble fibroblasts [9].

Valvular cells and their reciprocal interactions are essential players in CAVD [11]. The mammalian heart built of cardiomyocytes also contains a large population of non-myocyte cells (e.g., interstitial cells, endothelial cells) [12,13]. Recently, VICs have been discussed in the literature as important players in the degeneration of the heart valves, participating in the pathogenesis of CAVD [11,14,15,16]. These double-faced cells seem to be crucial in homeostasis regulation but, on the other hand, are responsible for aortic valve fibrosis and osteogenic degeneration, which is associated with activation of developmental transcriptional regulatory pathways [17,18,19,20]. Of note is that aortic valve cells populating particular cusps may have a distinct embryological origin, since LCC and RCC arise from the conotruncal (superior and inferior septal) cushions, while NCC arises from the posterior intercalated cushion of the outflow tract [10,21]. This may explain important functional differences in VICs isolated from different cusps [22].

According to the literature, VICs constitute a heterogenous population and can be divided into several phenotypes, which are engaged in physiological and pathological processes. Liu et al. [16] distinguished five phenotypes in the VICs family: embryonic progenitor endothelial/mesenchymal cells, quiescent VICs (qVICs), activated VICs (aVICs), progenitor VICs (pVICs), and osteoblastic VICs (obVICs). The cytological features of activated VICs (aVICs) correspond to those of myofibroblasts, described in valves by numerous authors and reported to play an important role in valve fibrosis contributing to degenerative processes such as valve sclerosis and atherosclerosis [23]. However, Combs et al. claimed that neither the commitment of VICs to the separate, fixed lineages nor the specific origin of VIC in distinct valve compartments have been unequivocally defined and proved [24].

It has been found that in the heart, there exists a population of interstitial cells morphologically similar to interstitial Cajal-like cells/telocytes (TCs) [25,26,27,28,29,30,31]. Cardiac interstitial Cajal-like cells have a mesenchymal origin, make numerous contacts with other types of surrounding cells, as well as with each other. They are involved in the 3D-tissue organization and fibrosis, tissue remodeling, and repair [32,33,34,35,36], as well as, indirectly, in the organization of the extracellular matrix, structural support, creation of microenvironments, intercellular communication, neurotransmission, immunomodulation and immunosurveillance, inhibition of apoptosis, and control, regulation and source of other cell types [37].

Recently it has been suggested that also in heart valves, there exists a population of cells expressing both hematopoietic and mesenchymal stem cell markers akin to interstitial Cajal-like cells/telocytes (TCs), which could constitute a special subpopulation of the VICs [35].

The aim of this study was to examine the presence, distribution, and morphology of cells co-expressing CD34 and PDGFRα in human aortic valves, and to investigate if the presence of these cells is associated with the occurrence of the early signs of valve calcific degeneration.

## 2. Results

### 2.1. CD34+/PDGFRα+ Cells Constitute Substantial Subpopulation of VICs

**Figure 1:**
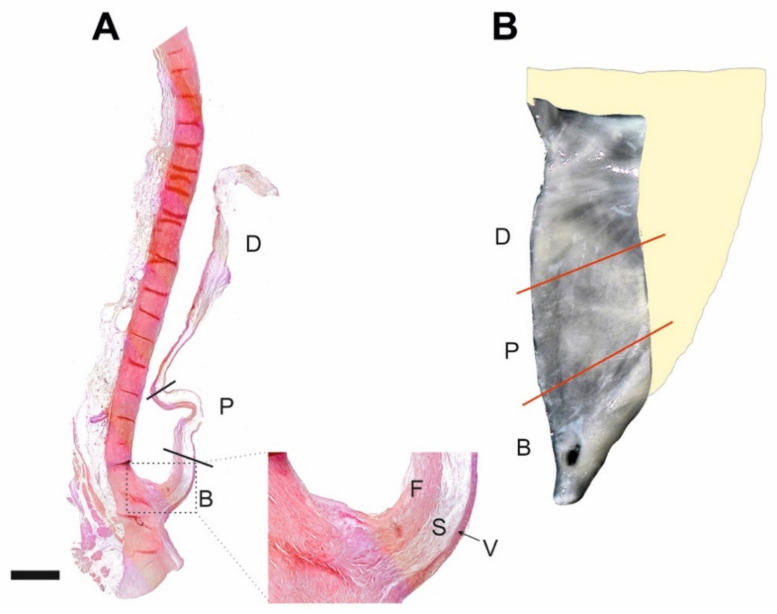
Normal morphology of the aortic valve. (**A**) Paramedian longitudinal section of a valve cusp spanning its basal–B, proximal–P, and distal–D parts. Higher magnification of the basal cusp part (inset) shows typical three-layered architecture: F–fibrosa, S–spongiosa, V–ventricularis. Hematoxylin and eosin staining. Scale bar = 2 mm. (**B**) Stereomicroscope image showing whole-mount specimen of the medial part of the bisected cusp with demarcation of its three parts (B, P, D) as in (**A**).

**Figure 2:**
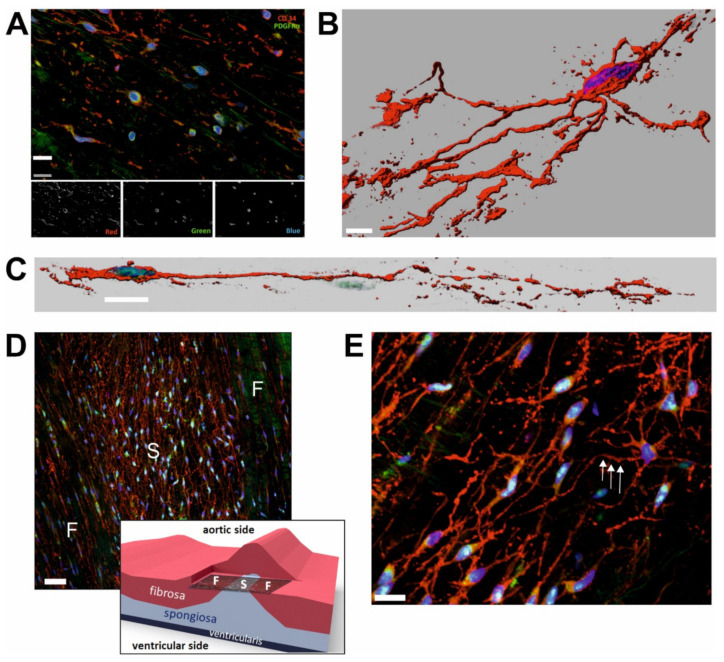
Identification, variability, and spatial organization of CD34/PDGFRα double positive cells in the human aortic valve. (**A**) Laser scanning confocal microscopy image showing co-localization of CD34 (red) and PDGFRα (green) in selected cells. Grayscale images show signals from the red, green, and blue channels, respectively. (**B**,**C**). Reconstruction images of two morphologically different CD34+/PDGFRα+ cells based on the whole-mount valve specimens imaged by confocal microscopy and subsequently rendered using the Imaris (Bitplane AG, Zürich, Switzerland) software. (**D**) Differences in the spatial organization of CD34+/PDGFRα+ cells in fibrosa-F, and spongiosa-S. Whole-mount specimen. Imaged cusp area is presented graphically. (**E**) High magnification image showing three-dimentional network of CD34+/PDGFRα+ cells in spongiosa. Note the moniliform projections (one is arrowed). Whole-mount specimen. Scale bars: 15 µm (**A**), 10 µm (**B**,**C**), 50 µm (**D**), 20 µm (**E**).

All examined valves were pliable and macroscopically normal. Histological examination revealed their well-preserved, three-layered structure (Figure 1). Cells double positive for CD34 and PDGFRα (Figure 2) were found in the fibrosa and spongiosa layers of all the examined valves and their density in the individual valves ranged from 20 to 290/mm^2^, i.e., from 5% to 63% of the whole VICs population. As the other VICs, they were also positive for vimentin.

### 2.2. Morphology and Spatial Organization of CD34+/PDGFRα+ Cells Is Regionally Different

The whole-mount specimens, z-stack imaged by confocal microscopy, allowed to visualize diverse morphology of CD34+/PDGFRα+ cells: from oval, without any projections, to much more complex forms with numerous branched, slender, frequently moniliform projections tens to hundreds of micrometers long (Figure 2) arising from relatively small cell bodies. Moreover, these cells were characterized by different spatial distributions in the valve layers. In fibrosa, they were mostly parallel to the collagen fibers, highly elongated or spherical, while in spongiosa, they were organized into three-dimensional net-like structure (Figure 2D).

### 2.3. The Abundance and Morphology of CD34+/PDGFRα+ Cells Are Associated with Age

**Figure 3:**
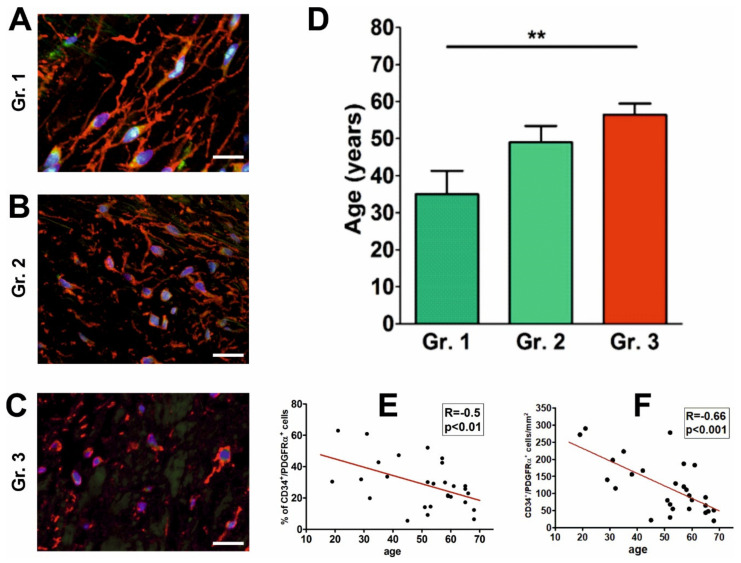
Changes in morphology and abundance of CD34+/PDGFRα+ cells related to age. (**A**–**C**) Confocal microscopy images of CD34+/PDGFRα+ cells representative for the examined valve groups: 1, 2, and 3, respectively. Note, gradual reduction of cell projections comparing Group 1 to Groups 2, and 3. Scale bars = 20 µm. (**D**) Graph showing the mean age of the valve donors in three groups of aortic valves: Group 1 valves were from significantly younger subjects than Group 3 valves (** *p* < 0.01; values presented as the means ± SD). Graphs (**E**,**F**) demonstrate a significant decrease in the CD34+/PDGFRα+ cell percentage and number, respectively, with age.

On the basis of the prevalence of morphological forms of CD34+/PDGFRα+ VICs, we distinguished three groups among all examined aortic valves. Group 1 (*n* = 5; mean age 35 ± 6.03)—cells with numerous and long projections, group I2 (*n* = 8; mean age 49 ± 4.42)—cells with short unbranched projections, group 3 (*n* = 15; mean age 56.5 ± 3.01)—cells with virtually no projections (Figure 3A–C).

Our results showed that valves with morphologically most complex CD34+/PDGFRα+ VICs (group I) were derived from significantly younger individuals (Figure 3D). Moreover, both the number and percentage of CD34+/PDGFRα+ VICs were inversely correlated with the age of the subjects (R = −0.66; *p* < 0.001 and r = −0.5; *p* < 0.01 for number and percentage, respectively) Figure 3E,F.

### 2.4. Early Calcification Is Associated with Decrease in the Number and Proportion of CD34+/PDGFRα+ Cells

**Figure 4:**
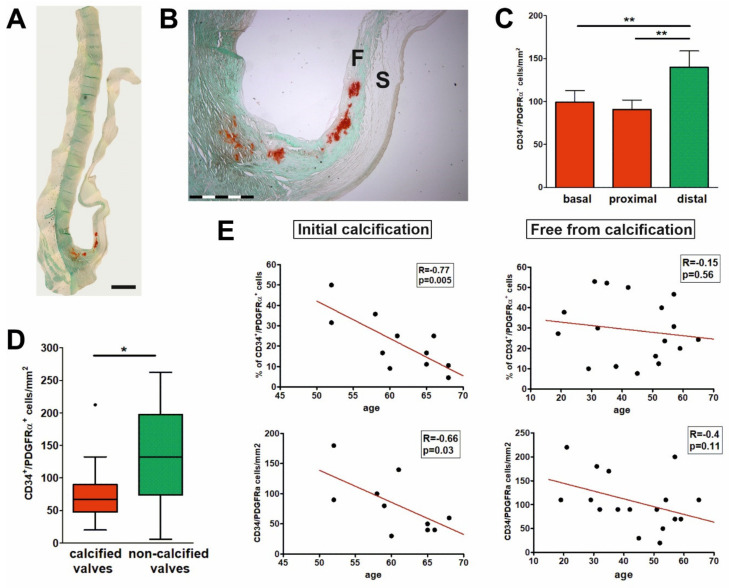
Association of CD34+/PDGFRα+ cells with initial calcification in aortic valves. (**A**) Micrograph of Alizarin red-stained non-stenotic aortic valve with signs of calcification (red) in the basal part of the cusp. As seen at higher magnification (**B**) calcifications are limited to the fibrosa layer (F–fibrosa, S–spongiosa). Scale bars: 2 mm (**A**), 1.5 mm (**B**). (**C**) Density of CD34+/PDGFRα+ VICs in three parts of the valve: they are significantly more numerous in the distal parts (not affected by calcification) of the cusps (** *p* < 0.01; values presented as the means ± SD). (**D**) Graph showing that CD34+/PDGFRα+ cells in fibrosa are significantly less numerous in the calcified valves compared with non-calcified ones. Box-and-whisker plot. Whiskers indicate 1.5 × IQR (interquartile range) beyond the box (Tukey style). Dot indicates an outlier (* *p* < 0.05). In spongiosa, the difference was not significant (not shown). (**E**) Graphs demonstrating a substantial age-related reduction of CD34+/PDGFRα+ cell population: cell percentage (top) and density (bottom) in the basal/proximal fibrosa of the valves with calcifications in these areas (left panel) versus insignificant changes in the same valve areas of the valves free from calcifications (right panel).

CD34+/PDGFRα+ VICs were significantly less numerous in the basal and proximal parts of the cusps (99.26 ± 13.58; 90.74 ± 11.02; 140 ± 18.78 cells/mm^2^ for basal, proximal, and distal parts, respectively), i.e., areas prone to developing degenerative changes, especially calcifications (Figure 4C). We compared the cellular architecture of the aortic valves with and without signs of calcification. Early degenerative lesions (foci of calcifications in the basal and proximal parts of fibrosa), not affecting the general valve morphology (Figure 4A,B) were found in 11 cases (39%). These valves were from older individuals (mean age 61.27 ± 5.75 years vs. 43.53 ± 13.94 years for calcified and non-calcified valves; *p* < 0.001). Valves with calcifications contained significantly lower numbers of CD34+/PDGFRα+ cells in both, fibrosa (6.67 [4.75–9.00] vs. 13.25 [7.37–19.75] for calcified and non-calcified, respectively *p* < 0.05) (Figure 4D) and spongiosa (7.00 [4.33–13.50] vs. 16.00 [6.00–19.21] for calcified and non-calcified, respectively; not significant). A substantial reduction of CD34+/PDGFRα+ VICs population correlated with age (R = −0.77; *p* = 0.005 for percentage; R = −0.66; *p* = 0.03 for density) was seen only in the basal/proximal fibrosa of the valves with calcifications as compared with the valves without calcifications in these areas (Figure 4E).

## 3. Discussion

Interstitial cells constitute a significant cellular component of the human heart [12]. Their role in valves is essential for maintaining local homeostasis and pathologic remodeling. Demonstration of interstitial Cajal-like cells (telocytes) in various organs has attracted a lot of attention from the scientific community in the last decade. Irrespectively of telocyte diversity, CD34/PDGFRα seems to be a valuable marker used for their immunohistochemical identification in various localizations, including heart tissues [29,38]. In 2014, Yang et al. [35] were the first to demonstrate Cajal-like/telocyte cells different from the “traditional” VICs in human aortic valves. They were immunopositive for CD34/vimentin, CD34/c-kit, and CD34/PDGFRβ. In the following year, Zhou et al. [34] described CD34/PDGFRα positive cells in the valves of rats and humans.

Data on the abundance of the Cajal-like cells/telocytes in different cusp regions are scanty and difficult for comparison. The matter is complicated by the fact that their distribution in particular organs is not uniform [28]. Anyway, it could be supposed that Cajal-like cells/telocytes vary in different organs, usually constituting a few percent of the total cell number [39,40].

We found that CD34+/PDGFRα+ cells make up from 5% to 63% of the whole VICs population in the human aortic valves. In vitro studies on rat cardiac interstitial cells showed that CD34+/PDGFRα+ cells constituted 29–41% of the entire cell population [34]. In contrast, Popescu et al. [12] estimated the percentage of telocytes in the human cardiac interstitial tissue to be only 1%. Interestingly, they found a decrease in the number of these cells in aging hearts, which is consistent with the inverse correlation between aortic valve CD34+/PDGFRα+ cell number and age found in the present study.

The microarchitecture of the normal aortic valve is surprisingly complex, ensuring its strength and durability, but undergoing substantial disorganization in pathologic conditions [41,42]. The differences between valve layers include not only their chemical composition but also the directional arrangement of their components. Collagen fibers, being the principal component of the fibrosa mostly show a circumferential orientation in each cusp, while the elastic elements in the ventricularis generally are directed radially [43]. In spongiosa, joining the layers mentioned above, the predominant extracellular matrix (ECM) elements (proteoglycans and glycoproteins) form a fine, molecular network-like structure.

We found CD34+/PDGFRα+ VICs in the fibrosa and spongiosa layers. Analysis of the whole-mount specimens allowed us to visualize the different spatial organization of these VICs, reflecting the organization of ECM in those layers—highly elongated double-positive VICs, showing a parallel arrangement along the collagen fibers in fibrosa and the cellular meshwork in spongiosa.

Several lines of evidence suggest regional heterogeneity of both VICs and VECs in heart valves [44,45,46]. In this study, we observed that the density of CD34+/PDGFRα+ VICs is significantly lower in more proximal parts of the cusps compared to more distal ones. It is known that the proximal part of the cusp is more prone to develop degenerative changes; local calcifications and focal accumulations of inflammatory cells [6]. Furthermore, a significantly lower number of CD34+/PDGFRα+ VICs was found in the fibrosa of the valves with initial calcifications, i.e., in the layer, mostly as first affected by calcification.

It is known that valve structure evolves over the lifetime [47,48]. However, it is difficult to determine which valvular changes reflect natural adaptation to the hemodynamic conditions and which are merely degenerative. A decrease in the overall valve cellularity with age could be regarded as a naturally occurring phenomenon since it is known that valve aging is associated with decrease in valve cellularity [49]. Of note is that our results show not only a decrease in the number of CD34+/PDGFRα+ cells but also in their proportion to other VICs. Since this imbalance was particularly evident in initially calcified valves, it might suggest that CD34+/PDGFRα+ VICs are either very sensitive for calcific matrix remodeling or are important for maintaining local milieu resistant to calcification. It is also possible that both hypotheses are reasonable. Moreover, it is well recognized that dying VICs facilitate calcification [50,51,52]. It cannot be excluded that the increased rate of cell death (including CD34+/PDGFRα+ cells) in valve areas with lower cellularity creates per se local milieu prone to calcification.

An unexpected finding in this study was the morphological diversity of CD34+/PDGFRα+ VICs. Only a fraction of this cell population revealed typical telocyte morphology with long, moniliform projections. The other CD34+/PDGFRα+ VICs either had shorter, often unbranched processes or even almost no projections. This brings the question of whether such cells can be classified as an unusual subset of telocytes. It must be clearly stated that our study based on immunofluorescence and confocal microscopy of the whole-mount specimens and valve sections does not allow to answer this question. However, Richter et al. [53] described telocytes with extremely short processes in failing human heart tissue morphologically resembling our Group 3 CD34+/PDGFRα+ VICs. The authors attributed the significant reduction in the number of telocytes and the reduction or even absence of their processes (telopodes) to alterations of the ECM, especially to fibrosis. Therefore, it seems probable that all CD34+/PDGFRα+ VICs belong to the telocyte family and the gradual reduction of their projections with age found in the present study is associated with age-dependent changes of the valve interstitial microenvironment.

Different shapes of the body and projections of CD34+/PDGFRα+ VICs might be a result of aging accompanied by degenerative processes. For instance, telocytes described previously in the human heart are highly sensitive to hypoxia. They show severe ultrastructural alterations such as cytoplasmic vacuolization, absence of the telocytes labyrinthine components, as well as shrinkage and shortening of the telopodes (extensions) [53,54,55]. The milieu prone to hypoxia occurring in valves during age-related fibrocalcific remodeling might trigger a simplification of the form and reduction of the number of CD34+/PDGFRα+ VICs.

It remains unclear whether the existence of different morphological forms of CD34+/PDGFRα+ VICs in the human aortic valves reflects some pathological state, but the prevalence of their particular forms in certain age groups is a fact. Moreover, the number and percentage of CD34+/PDGFRα+ VICs inversely correlated with age. Thus, our older patients had a lower number of CD34+/PDGFRα+ VICs, among which simplified, “rudimentary” forms with significantly reduced projections were predominant. On the contrary, the younger population had a higher number and percentage of the cells with long-branched cellular projections. In our opinion, the population of CD34+/PDGFRα+ VICs could represent a “private reserve” of abilities to save a right balance in the local homeostasis. Besides, the same trend was observed when we compared calcified and non-calcified aortic valves—calcification was accompanied by decline of CD34+/PDGFRα+ VICs density in the tissue. It remains in agreement with results of Nomura et al. [56] who showed that a loss of the CD34 antigen positivity in VICs facilitated valvular ECM calcification.

Since our study is retrospective and comprises a relatively low number of cases, we cannot definitively determine if or which CD34+/PDGFRα+ VICs changes precede or follow the calcific remodeling.

According to the VIC paradigm, the main cells in the valve stroma are responsible for the maintenance of valve ECM, and the balance between their quiescent and activated phenotype is fundamental for valve homeostasis [16,57]. Most of them, in normal conditions, are classified as quiescent VICs (qVICs). They preserve the natural three-layered, avascular valve morphology continuously renewing defective ECM components, in particular collagen, what may cause their transient activation (aVICs) by various mechanical (hemodynamic) and biochemical stimuli [58]. However, prolonged activation and proliferation of myofibroblast-like activated VICs are associated with aberrant remodeling of the valve with its fibrosis, calcification, and occasionally ossification driven by VICs with osteogenic potential (obVICs).

It is known that VICs include a subpopulation of progenitor cells (pVICs) comprising resident valvular cells and circulating (bone marrow-derived) cells entering valve stroma. The occurrence of progenitor cells expressing both endothelial and mesenchymal markers in mature valves supports the thesis that in adults, the process of endothelial to mesenchymal transition (EMT), fundamental in valvulogenesis also supplements the VICs population [59]. It is not clear if the differences in the origin of VICs influence their activity in physiologic and pathologic conditions. Some evidence suggests the participation of EMT in the calcific remodeling of the valves. Hjortnaes et al. [57] demonstrated that EMT precedes calcification and transition of VECs into osteoblastic-like cells in aortic valves. Interestingly, they also found that VICs may suppress EMT and calcification of VECs undergoing EMT.

Recent studies indicate that PDGFRα positive cells in various localizations may participate in either proper tissue regeneration after injury or aberrant, fibrotic remodeling, depending on their differentiation status [60,61]. Bearing in mind that CAVD is an age-related pathology affecting mostly older subjects, the decreased percentage and number of CD34+/PDGFRα+ VICs in elder individuals as well as in valves subjected to initial calcification suggests that this VICs subpopulation is only indirectly involved in calcific degeneration.

On the other hand, CD34+/PDGFRα+ VICs with long projections, resembling typical Cajal-like cells/telocytes, being more numerous in younger subjects, could be involved in the maintenance of intravalvular structural (3-dimensional, layered organization of ECM) organization. Moreover, some data suggest that interstitial Cajal-like cells/telocytes in the lungs might be involved in the modulation of oxidative stress levels and the inhibition of apoptosis, both being well-established factors participating in calcific aortic valve degeneration from its earliest stages [62,63,64].

In conclusion, we have demonstrated that normal aortic valves contain a subpopulation of VICs co-expressing CD34 and PDGFRα, which might be involved in the maintenance of the local microenvironment resistant to pathologic remodeling. Their lower number and reduced processes in older age may substantially limit self-regenerative properties of the valve stroma, making it more prone to degeneration and calcification. Further studies are required to analyze more data and parameters of valve interstitial cells. Nevertheless, the new subpopulation of VICs, double-positive for CD34 and PDGFRα, should be taken into account for a better understanding of the pathogenesis of the most widespread human heart valve pathology.

## 4. Materials and Methods

### 4.1. Subjects

Aortic valve specimens were obtained upon autopsy from 28 subjects (mean age 50.50 ± 14.33 years)—twenty-two men (79%) and six women (21%), who died accidentally. No cardiovascular pathologies were detected upon autopsy. Following a gross inspection under Stemi 2000C stereomicroscope (Zeiss, Jena, Germany), all cusps were bisected along the line passing from the cusp base to its free margin and the left half of each was subjected to the routine histological procedure, while the right half was processed as a whole-mount (WM) specimen in order to show the complex three-dimensional structure of VICs (Figure 1). To avoid the influence of intercuspal differences [46], non-coronary cusps (usually calcified at the earliest) were subjected to quantitative evaluation.

### 4.2. Ethical Approval

The study was conducted in accordance with the moral, ethical, regulatory and scientific principles governing clinical research. All samples were retrieved with the approval of the Jagiellonian University Bioethical Committee using procedures that conformed to the Declaration of Helsinki guidelines (approval number–1072.6120.103.2018; 20 April 2018).

### 4.3. Tissue Processing

All tissue samples were fixed for 24 h in 4% buffered paraformaldehyde. For routine histology, six µm serial sections were cut and mounted on polylysine-coated slides (Menzel-Glaser; Thermo Scientific, Dreieich, Germany). The sections were deparaffinized, rehydrated, and stained either with hematoxylin-eosin (HE) to assess general valve morphology or with Alizarin red for detection of calcified areas.

### 4.4. Immunofluorescence

After deparaffinization and rehydration, the slides were preincubated for 40 min with 5% normal goat serum (Vector, Burlingame, CA, USA; # S-1000) in PBS containing 0.01% sodium azide, 0.05% thimerosal, 0.1% bovine serum albumin and 0.5% Triton X-100 to reduce nonspecific binding and to increase penetration of the antibodies. Then specimens were incubated at 4 °C either overnight or for 1.5 days with gentle rotation in case of whole-mounts in PBS with the normal serum containing a primary antibody (or a mixture of primary antibodies) and 0.5% Triton X-100. After five washes (10 min each) in PBS, the specimens were incubated for 1.5 h (overnight in case of whole-mounts) at room temperature with the secondary antibody (or a mixture of secondary antibodies). Indirect double immunofluorescence for identification of VIC, both in sections and whole- mounts were performed with mouse anti-human or rabbit anti-human primary antibodies against CD34 (1:50; code: NCL-END; Novocastra, Newcastle, UK/Leica Biosystems, Newcastle upon Tyne, UK), PDGFRα (1:100, code: ab124392; Abcam, Cambridge, UK) and vimentin (1:100, code: NCL-VIM; Novocastra, Newcastle, UK/Leica Biosystems, Newcastle upon Tyne, UK). Secondary antibodies included goat anti-mouse Alexa555-conjugated antibody (1:200; code: A-21424; Molecular Probes, Eugene, OR, USA) and goat anti-rabbit Alexa488-conjugated antibody (1:200; code: A-11008; Molecular Probes, Eugene, OR, USA). Negative controls were performed with the omission of the primary antibodies. Finally, the slides were washed in two changes (10 min each) of PBS and coverslipped in fluorescence mounting medium containing DAPI (4’,6-diamidino-2-phenylindole) for counterstaining of cell nuclei (Sigma, Saint Louis, MO, USA).

### 4.5. Microscopic Examination

Specimens were examined under a scanning confocal microscope (FluoView FV1200, Olympus, Tokyo, Japan). The images were collected using the FV10-ASW v.4.2a (Olympus, Tokyo, Japan) software. The number of cells in the respective part/layer was counted in 5–10 randomly chosen high magnification (400×) fields and recalculated for square millimeters. In order to show the complex three-dimensional structure of the immunolabelled VICs, whole-mount valve specimens were z-stack imaged by confocal microscopy and subsequently rendered using the Imaris v. 7.7.1 (Bitplane AG, Zürich, Switzerland) software. All samples were assessed by two independent observers (each blinded to the other) without any knowledge of the clinical parameters to avoid bias.

### 4.6. Statistical Analysis

The variables were expressed as mean ± SD or median [lower-upper quartile values] depending on their type and distribution. The statistical analysis included (in compliance with the specific category of collected data) *t*-Student test (or Mann–Whitney U test) and repeated measures ANOVA test followed by the Newman–Keuls post hoc test. Correlations between continuous variables were analyzed by the Pearson test. Statistical analyses were performed using the Statgraphics Centurion XVI (StatPoint Technologies INC, Warrenton, USA) software. All tests were two-tailed with *p* < 0.05 considered statistically significant.
